# Astrocytic Control of Biosynthesis and Turnover of the Neurotransmitters Glutamate and GABA

**DOI:** 10.3389/fendo.2013.00102

**Published:** 2013-08-15

**Authors:** Arne Schousboe, Lasse K. Bak, Helle S. Waagepetersen

**Affiliations:** ^1^Department of Drug Design and Pharmacology, Faculty of Health and Medical Sciences, University of Copenhagen, Copenhagen, Denmark

**Keywords:** glutamate, GABA, astrocyte, neurotransmitter, homeostasis, energy

## Abstract

Glutamate and GABA are the quantitatively major neurotransmitters in the brain mediating excitatory and inhibitory signaling, respectively. These amino acids are metabolically interrelated and at the same time they are tightly coupled to the intermediary metabolism including energy homeostasis. Astrocytes play a pivotal role in the maintenance of the neurotransmitter pools of glutamate and GABA since only these cells express pyruvate carboxylase, the enzyme required for *de novo* synthesis of the two amino acids. Such *de novo* synthesis is obligatory to compensate for catabolism of glutamate and GABA related to oxidative metabolism when the amino acids are used as energy substrates. This, in turn, is influenced by the extent to which the cycling of the amino acids between neurons and astrocytes may occur. This cycling is brought about by the glutamate/GABA – glutamine cycle the operation of which involves the enzymes glutamine synthetase (GS) and phosphate-activated glutaminase together with the plasma membrane transporters for glutamate, GABA, and glutamine. The distribution of these proteins between neurons and astrocytes determines the efficacy of the cycle and it is of particular importance that GS is exclusively expressed in astrocytes. It should be kept in mind that the operation of the cycle is associated with movement of ammonia nitrogen between the two cell types and different mechanisms which can mediate this have been proposed. This review is intended to delineate the above mentioned processes and to discuss quantitatively their relative importance in the homeostatic mechanisms responsible for the maintenance of optimal conditions for the respective neurotransmission processes to operate.

## Introduction

### Historical perspective of amino acid neurotransmission: Glutamate and GABA

**Figure 1:**
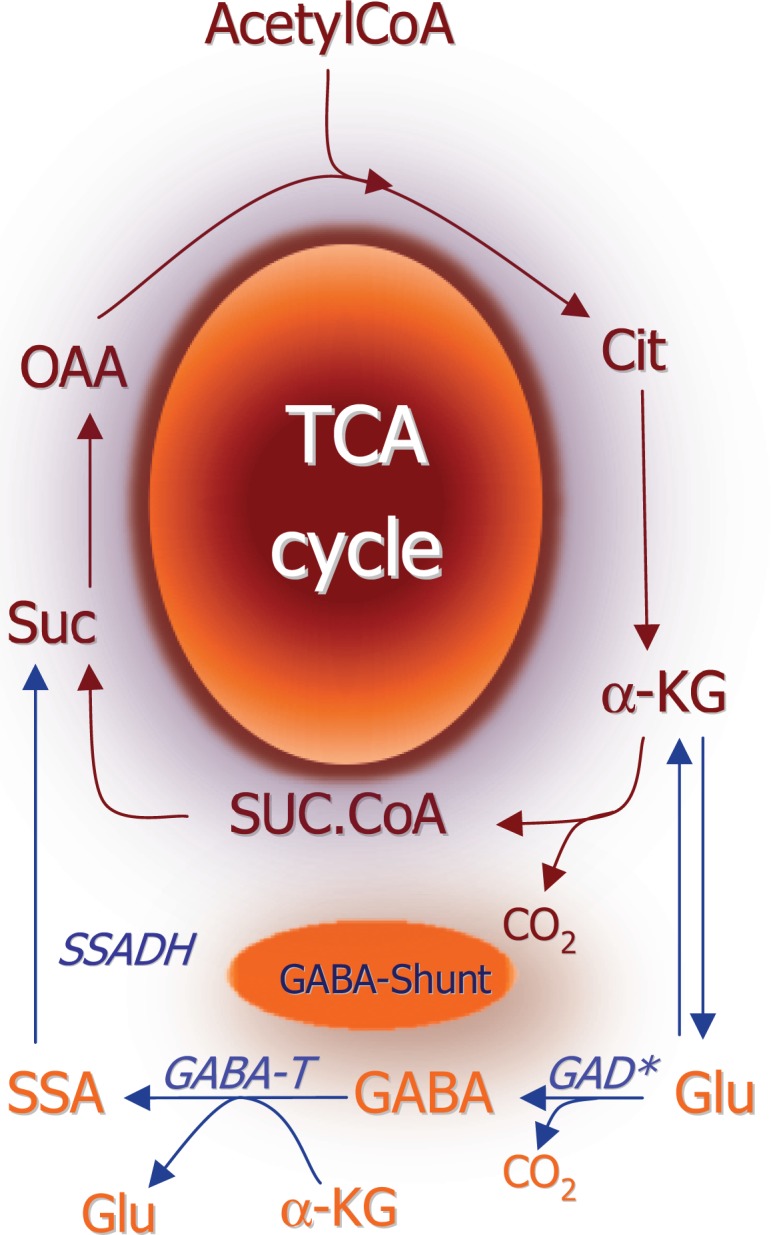
**Schematic representation of the TCA cycle and the GABA shunt which provides an alternative pathway for conversion of αKG to succinate**. (*) Please note that glutamate decarboxylase is localized in the cytosol whereas all other enzymes depicted are mitochondrial. αKG, α-ketoglutarate; SSADH, succinate-semialdehyde; GABA-T, GABA-transaminase; GABA, γ-aminobutyrate; GAD, glutamate decarboxylase; Glu, glutamate.

The general concept in biochemistry that glutamate is a key metabolite linking amino acid and glucose metabolism via the tricarboxylic acid (TCA) cycle was extended when Eugene Roberts discovered GABA in the brain. They showed that it was synthesized from glutamate by decarboxylation of the C-1 carboxylic group thereby adding an important functional property to glutamate (1). Further studies of glutamate and GABA metabolism led to the biochemical term “the GABA shunt” (2) which interconnects the TCA cycle with glutamate and GABA metabolism (Figure 1). While the basic biochemical metabolic pathways (see below) involving glutamate and GABA metabolism were worked out from 1950 and onward it was not until the end of this decade that pioneering electrophysiological studies of spinal cord neurons led to the understanding that glutamate and GABA act as excitatory and inhibitory substances (3, 4). Due to the fact that these amino acids were present in much higher concentrations than the classical neurotransmitters acetylcholine and the monoamines it took several years until it was generally accepted that these two amino acids are not just metabolites but they constitute the quantitatively most important neurotransmitters in the central nervous system [see reviews by Ref. (5) and (6)].

## Content

### Biosynthetic pathways for transmitter glutamate and GABA

#### Glutamate

It is commonly accepted that the enzyme catalyzing the hydrolysis of glutamine to glutamate, glutaminase is the functionally most important enzyme for the biosynthesis of glutamate [see Ref. (7)] and since this enzyme is activated by phosphate it is routinely referred to as phosphate-activated glutaminase (PAG). This enzyme was first described by Krebs (8) in an extensive study of glutamine degradation and synthesis in different tissues including the brain. The expression of PAG is higher in neurons than in astrocytes (9, 10) but PAG is not specifically associated with glutamatergic neurons as GABA synthesis in GABAergic neurons is dependent upon glutamate derived from glutamine taken up from surrounding astrocytes (see below).

There are actually alternative enzymatic reactions which are able to produce glutamate from the TCA cycle intermediate α-ketoglutarate. These are glutamate dehydrogenase (GDH) and the aminotransferases. Among the latter aspartate aminotransferase (AAT), alanine-aminotransferase (ALAT), and the branched chain aminotransferase (BCAAT) are the most likely candidates to be involved in glutamate biosynthesis [for reactions and references, see Ref. (11)]. While the aminotransferase reactions would normally be in thermodynamic equilibrium the GDH reaction in the brain is unlikely to be at thermodynamic equilibrium due to low levels of ammonia and NADH/NADPH (12). The *K*_m_ for ammonia in the GDH-catalyzed reaction is in the millimolar range, i.e., well above the level of ammonia found in the brain; however, in a mitochondrial microenvironment close to the PAG reaction in glutamatergic neurons, the level of ammonia might be high enough for the reaction to proceed in the direction of reductive amination as initially suggested by Waagepetersen et al. (13) and discussed by Bak et al. (14). In the liver, however, where the substrate conditions are different the GDH reaction may well be at thermodynamic equilibrium (15). As pointed out by Cooper (12) the GDH-catalyzed reaction is often coupled to the aminotransferases, which in the brain would result in ammonia being produced from the amino group in the amino acids since the GDH reaction would preferentially catalyze oxidative deamination of glutamate. This aspect will be discussed in more detail below.

**Figure 2:**
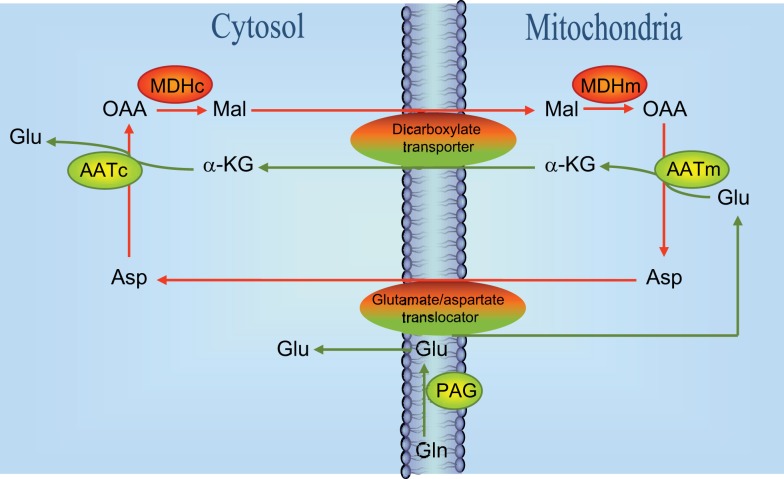
**Schematic drawing of the reactions involved in the biosynthesis of neurotransmitter glutamate**. Reactions involve the elements of the malate-aspartate-shuttle (43), i.e., the glutamate/aspartate translocator and the dicarboxylate transporter as indicated. Notice that a significant fraction (about 50%) of glutamate derived from the reaction catalyzed by phosphate-activated glutaminase (PAG) is directly entering mitochondria presumably via the glutamate/aspartate translocater. It has also been suggested that PAG is catalytically active in the mitochondrial matrix (23, 24) although this is controversial [see text and Ref. (22)]. MDHc, cytosolic malate dehydrogenase; MDHm, mitochondrial malate dehydrogenase; AATc, cytosolic aspartate aminotransferase; AATm, mitochondrial aspartate aminotransferase; OAA, oxaloacetate; Mal, malate; Glu, glutamate; Asp, aspartate; GLN, glutamine; α-KG, α-ketoglutarate.

Based on experiments performed in cultured glutamatergic cerebellar granule cells using phenylsuccinate to inhibit the ketodicarboxylate carrier in the mitochondrial membrane (16) a model was proposed according to which (Figure 2) neurotransmitter glutamate is generated by the concerted action of glutaminase and the malate-aspartate-shuttle (MAS). According to this model glutamate formed by the action of PAG located in the mitochondrial membrane (17) gets access to the mitochondrial matrix where it is transaminated by AAT to form α-ketoglutarate which is translocated to the cytoplasm where it undergoes a second transamination via AAT to produce glutamate. That this mechanism is indeed working is supported by the demonstration that while oxidation of the carbon skeleton of glutamate in the TCA cycle is dependent on activity of GDH (18), the formation of glutamate from α-ketoglutarate is catalyzed by an aminotransferase which is most likely mitochondrial AAT (19). This glutamate is subsequently used for vesicular release (16). The translocation processes require the operation of the aspartate-malate shuttle (see Figure 2). In keeping with this the release of transmitter glutamate could be inhibited not only by phenylsuccinate that inhibits the dicarboxylate carrier (20) but also by aminooxyacetic acid that inhibits AAT (20). It has been argued that glutamate generated in the PAG catalyzed hydrolysis of glutamine is released from the mitochondrial membrane into the cytosol and not into the matrix [see Figure 2; (17, 21, 22)] but experiments using [^13^C]glutamine and histamine to inhibit glutamine transport into the mitochondria have provided evidence that glutamate generated in the PAG catalyzed reaction does indeed get access to the mitochondrial matrix (23, 24).

#### GABA

While no single enzyme can be used as a specific marker for glutamatergic neurons (see above), the demonstration by Saito et al. (25) using an antibody which specifically recognizes the GABA synthesizing enzyme glutamate decarboxylase (GAD) that GAD is associated selectively with GABAergic neurons has led to the notion that GAD is a selective marker for GABAergic neurons. The subsequent finding of GAD-like immunoreactivity in certain glutamatergic neurons in the hippocampus (26, 27) may, however, indicate a more complex distribution of GAD the functional implications of which may not be easy to understand. It should be noted in this context that GABA not only acts as transmitter but also functions as a neurotrophic agent [see Ref. (28), and references herein]. Cloning studies have revealed that isoenzymes of GAD exist since two cytosolic forms of GAD with molecular weights of 65 and 67 kDa, respectively have been described [see Ref. (29) for review]. The general notion is that the intracellular distribution of the two isozymes is heterogeneous, since GAD_65_ is selectively associated with GABAergic nerve endings and therefore plays an important regulatory role in synthesis of neurotransmitter GABA (30). This is compatible with the observation that GAD_65_ knock-out (GAD_65_^−/−^) animals are susceptible to induced seizures (31, 32). GAD_67_ is found throughout the cytosol of these neurons and therefore may serve as the biosynthetic enzyme for the GABA pool associated with metabolism [i.e., consistent with a housekeeping function; see Ref. (33)]. It may be noted that GAD_67_ knock-outs (GAD_67_^−/−^) are lethal while heterozygotes are viable and have no seizure sensitivity contrary to the GAD_65_ knock-outs (31, 34).

**Figure 3:**
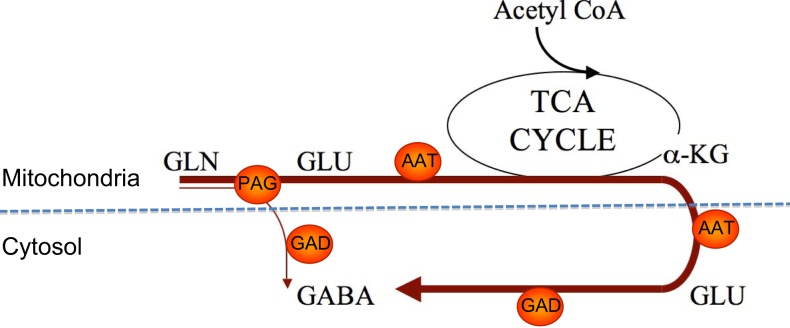
**An illustration of two pathways for GABA synthesis from glutamine**. The bold arrow illustrates the predominant pathway where the carbon skeleton of glutamine is metabolized via the TCA cycle prior to synthesis of GABA. The thin arrow illustrates the direct synthesis of GABA from glutamine without involvement of the TCA cycle. Note that AAT is present in both the cytosol and the mitochondrial matrix. αKG, α-ketoglutarate; PAG, phosphate-activated glutaminase; ATT, aspartate aminotransferase; GAD, glutamate decarboxylase.

As illustrated in Figure 3 the biosynthesis of neurotransmitter GABA may be somewhat more complex than originally thought of as studies of GABA synthesis from [^13^C]glutamine have revealed that the synthesis involves mitochondrial TCA cycle metabolism (35, 36). As pointed out above hydrolysis of glutamine by PAG leads to generation of glutamate in the mitochondrial matrix and this glutamate needs to be transaminated to form α-ketoglutarate in order for the carbon skeleton to leave the mitochondria (see Figure 2). Since GAD is a cytosolic enzyme (37) the decarboxylation of glutamate to form GABA can take place only in the cytosol. As a result of the fact that at least some of the glutamate generated in the PAG reaction may be released directly into the cytosol (17) GABA can be formed either directly from glutamate in the cytosol or from glutamate originating from α-ketoglutarate produced in the TCA cycle from oxidatively deaminated glutamate involving GDH. These two pathways seem to be almost equally important for biosynthesis of transmitter GABA (35, 36). The functional significance of the mitochondrial involvement in GABA biosynthesis is not well understood but it may provide additional possibilities for regulatory mechanisms to operate.

### Metabolic pathways for degradation of glutamate and GABA

While the primary event responsible for inactivation of the neurotransmitter action of glutamate and GABA is related to the high-affinity transporters for these amino acids, the subsequent intracellular metabolic pathways for degradation of glutamate and GABA are of functional significance [Ref. (38) and references herein]. A discussion of the transporters will follow below.

The enzymes of interest are glutamine synthetase (GS), GDH, and aminotransferases (or transaminases) in relation to glutamate. In case of GABA the pertinent enzymes are GABA-transaminase (GABA-T) and succinic semialdehyde dehydrogenase (SSADH). GS is a cytosolic enzyme while GDH, SSADH, and GABA-T are located in the mitochondria [Ref. (34) and references herein]. Except for GS which is strictly astroglial (39) these enzymes are expressed both in neurons and astrocytes. Transgenic or knock out animals of these enzymes (GS, GDH, and SSADH) have provided evidence that they are of significant, functional importance for the maintenance of homeostatic mechanisms related to the cellular contents as well as to the neurotransmitter function. Thus, GDH knock outs exhibit impaired astrocytic glutamate oxidation and increased glutamine synthesis pointing to a more prominent cycling of glutamate which may be compatible with the observation that synaptic glutamatergic function remained intact (40). On the other hand, GS homozygote knock outs are lethal and heterozygotes exhibit increased susceptibility to seizures (41). SSADH^−/−^ animals exhibit altered neurotransmitter GABA metabolism and increased seizure activity which may result in lethal status epilepticus [see Ref. (34)].

### Role of astrocytes providing glutamine, the precursor for glutamate and GABA

**Figure 4:**
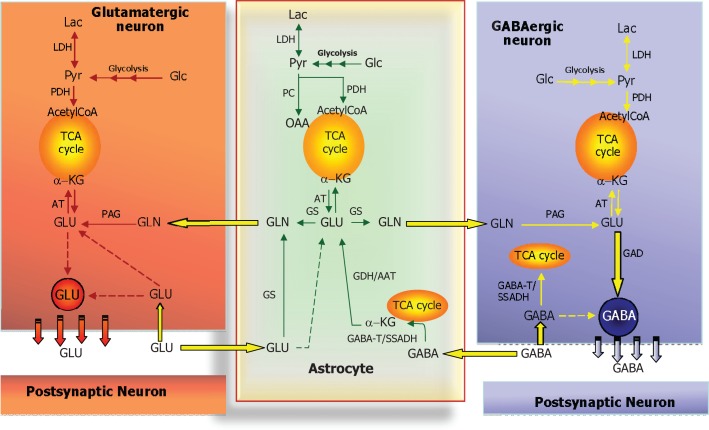
**Schematic cartoon depicting the metabolic interactions between a glutamatergic neuron, a GABAergic neuron, and a nearby astrocyte**. In all three cell types, glucose (Glc) is metabolized to pyruvate via the multi-step process of glycolysis and either reduced to lactate (by lactate dehydrogenase, LDH) or oxidized to acetyl-CoA (by pyruvate dehydrogenase complex, PDH) which will subsequently be oxidized in the TCA cycle. In astrocytes, pyruvate may also undergo carboxylation to form oxaloacetate (OAA), an anaplerotic reaction catalyzed by pyruvate carboxylase (PC). At the glutamatergic synapse, glutamate (GLU) released as neurotransmitter will be taken up by nearby astrocytes and amidated to glutamine (GLN) by glutamine synthetase (GS) and returned to the neuron for re-use as neurotransmitter, the so-called glutamate-glutamine cycle. In neurons, GLU is re-formed from GLN by the mitochondrial enzyme phosphate-activated glutaminase (PAG). A similar cycle exists at the GABAergic synapse; however, the carbon skeleton of GABA enters the TCA cycle as indicated. GABA is transformed to the TCA cycle intermediate succinate via two reactions catalyzed by GABA-transaminase (GABA-T) and succinic semialdehyde dehydrogenase (SSADH). The GABA-T catalyzed reaction produces GLU from α-KG that may then be used as precursor for GLN synthesis and eventual synthesis of GABA in the neuron. This process is known as the GABA shunt since it by-passes two reactions of the (astrocytic) TCA cycle. In GABAergic neurons, GABA is synthesized from GLU by the enzyme glutamate decarboxylase (GAD). Notice that in all three cell types, GLU is in transamination equilibrium (catalyzed by aminotransferases, AT) with α-KG linking TCA cycle metabolism with GLU and GABA homeostasis. Also notice that re-uptake of released GLU and GABA takes place to some extent as well and that the GABA shunt works in GABAergic neurons when GABA is taken up pre-synaptically.

**Figure 5:**
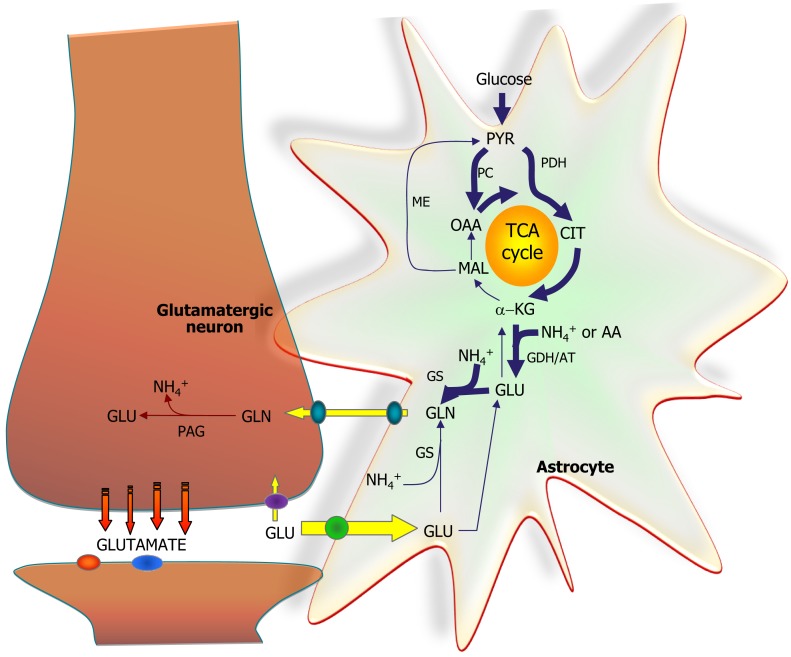
**The astrocytic part of the synapse provides a net synthesis of glutamine (GLN) via the concerted action of pyruvate carboxylase (PC) and pyruvate dehydrogenase (PDH) generating OAA and acetyl-CoA, the combination of which leads to synthesis of CIT**. This subsequently leads to a net synthesis of α-ketoglutarate (α-KG) allowing synthesis of glutamate (GLU) catalyzed by either glutamate dehydrogenase (GDH) or an amino acid aminotransferase (AA). Glutamate is used for synthesis of glutamine (GLN) catalyzed by glutamine synthetase (GS). Glutamine is transferred to the glutamatergic neuron to be used for synthesis of glutamate catalyzed by phosphate-activated glutaminase (PAG). Released glutamate is taken up into the astrocyte and transformed into glutamine completing the glutamate-glutamine cycle. Alternatively the glutamate taken up may be oxidatively metabolized which subsequently requires *de novo* synthesis of glutamine via the anaplerotic processes indicated in bold arrows.

**Figure 6:**
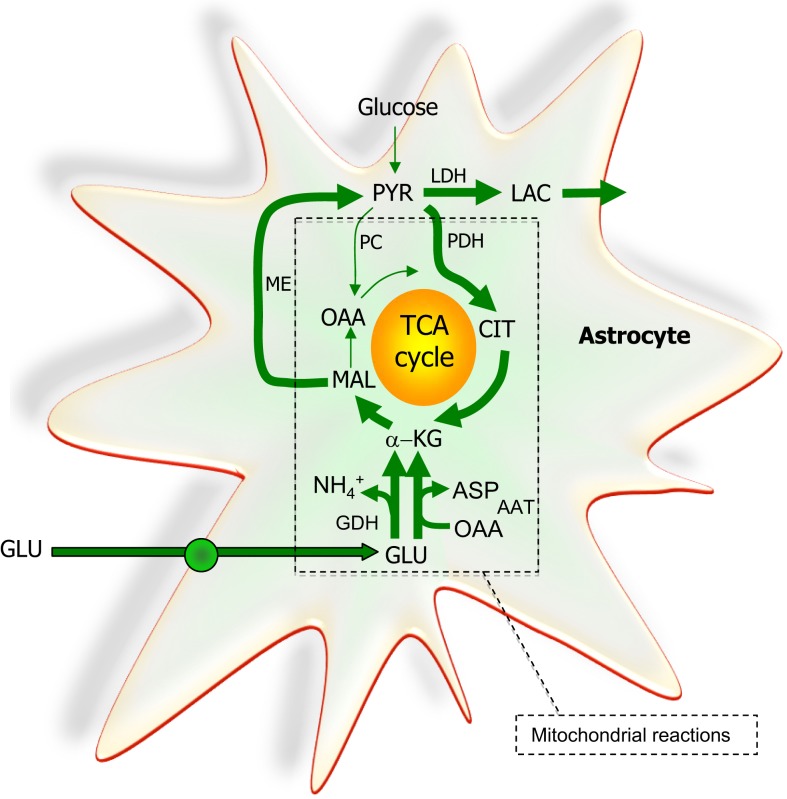
**The extent to which glutamate (GLU) is oxidized in astrocytes seems to increase particularly during higher glutamate concentrations**. A net synthesis of tricarboxylic acid (TCA) cycle intermediates occurs when the initial reaction is catalyzed by glutamate dehydrogenase (GDH) which paves the way for the complete oxidation of the carbon skeleton of glutamate. This requires pyruvate recycling via the concerted action of malic enzyme (ME) and pyruvate dehydrogenase (PDH) converting malate into acetyl CoA producing NAD(P)H. Acetyl CoA is oxidized completely in one turn of the TCA cycle. A partial oxidation of glutamate is acquired when pyruvate (PYR) is reduced to lactate (LAC) instead of being oxidized to acetyl CoA. The redox state of the cell is likely important in the regulation of the destiny of the glutamate molecule. As an alternative to the activity of GDH, aspartate aminotransferase (AAT) facilitates the formation of α-ketoglutarate (α-KG) from glutamate at the expense of oxaloacetate (OAA); thus no net synthesis of TCA cycle intermediates is obtained. In contrast to the complete oxidation initiated by the activity of GDH, AAT enables the truncated TCA cycle which refers to the net synthesis of aspartate from glutamate, a pathway shown to accelerate during hypoglycemic conditions. All enzymes except ME and LDH are located in the mitochondria. PC, pyruvate carboxylase; CIT, citrate.

Glutamine serves a very important role in glutamatergic and GABAergic neurotransmission, as it constitutes the only precursor for the biosynthesis of these two amino acid neurotransmitters (see above). Due to the fact that GS as stated above is exclusively expressed in astrocytes these cells play a unique role in supplying the precursor for the transmitter in both glutamatergic and GABAergic neurons [see Ref. (38) and references therein]. This requires a symbiotic relationship between the neuronal and astrocytic compartments since an exchange of glutamine and the transmitter amino acids is necessary. This is illustrated in Figure 4 which is a schematic representation of the glutamate/GABA-glutamine cycle operating between the nerve endings of glutamatergic or GABAergic neurons and surrounding astrocytes. This cycle was originally thought to operate in a stoichiometric fashion maintaining the carbon skeleton of the transmitter amino acids intact (42). However, the demonstration of a significant oxidative metabolism of both glutamate and GABA via the TCA cycle [see Ref. (43)] makes it impossible for this cycle to operate in a stoichiometric way as at least part of the glutamate and GABA skeleton will be lost as carbon dioxide. It is therefore imperative that such a loss is compensated for by *de novo* synthesis of the carbon skeleton of glutamate and subsequently glutamine. As ultimately the precursor must originate from the TCA cycle in the form of an intermediary which most likely is α-ketoglutarate (Figure 5) an anaplerotic mechanism must be operating involving *de novo* synthesis of oxaloacetate. In the brain this is most likely catalyzed by pyruvate carboxylase (PC) which has a higher activity than the other enzymes capable of synthesizing oxaloacetate, i.e., malic enzyme (ME) and phosphoenolpyruvate carboxykinase (44). Interestingly, PC is like GS an astrocytic enzyme (45, 46) and accordingly *de novo* synthesis of the carbon skeleton of glutamate and GABA via glutamine requires astrocytic participation [see Ref. (43, 47)]. It has been estimated that this anaplerotic, *de novo* synthesis of glutamine may account for replenishment of approximately 25–30% of the glutamate transmitter pool [see Ref. (43)]. It would be assumed, that this would be associated with a comparable complete oxidation of glutamate to carbon dioxide, a process requiring pyruvate recycling (Figure 6). It may therefore seem somewhat enigmatic that the activity of pyruvate recycling in the brain as well as in cultured brain cells appears relatively modest (43).

### Cellular distribution of glutamine transporters

As a consequence of the astrocytic localization of *de novo* synthesis of glutamine, a mechanism must exist which can mediate transport of glutamine out of the astrocytes and into glutamatergic and GABAergic neurons, respectively. This mechanism consists of a unique set of glutamine membrane transporters located either on astrocytes or the two sets of neurons. These transporters are referred to as System A transporters in neurons and System N transporters in astrocytes (14, 48). The System A family includes SNAT1, 2, and 4 and System N consists of SNAT3 and 5 (14). Each of these cloned transporters have alternative names making the nomenclature confusing [for a comprehensive review, see Ref. 38)]. These transporters are regulated in complex ways with regard to expression and kinetics underlining the functional importance of the transporters (38).

### Role of glutamate transporters in maintenance of the glutamate neurotransmitter pool, i.e., uptake of glutamate in glutamatergic neurons versus astrocytes

Detailed studies of the cellular localization of the most abundantly expressed glutamate transporters, GLAST (EAAT-1) and Glt-1 (EAAT-2) have provided compelling evidence that these glutamate transporters are expressed predominately in astrocytes (5, 49, 50). In addition to these astrocytic glutamate transporters, a neuronal counterpart (EAAC-1 or EAAT-3) has been cloned (51) and it has been proposed that if located in the plasma membrane of neurons, this transporter could participate in the maintenance of the pool of transmitter glutamate and GABA (52–54). However, a recent detailed study of the expression of EAAC-1 relative to that of GLT-1 and GLAST has shown that the expression level of the neuronal glutamate transporter EAAC-1 is very low and hence, it is enigmatic how this transporter can contribute significantly to removal of transmitter glutamate released from glutamatergic neurons thereby via re-uptake into the presynaptic nerve endings replenishing the transmitter pool of glutamate (55). Nevertheless, a functional study in glutamatergic neurons has shown that even in the presence of glutamine in the incubation medium, block of glutamate transport using the specific glutamate transporter inhibitor threo-benzyloxy-aspartate (TBOA) caused a significant decrease in the cellular glutamate content after repetitive release of glutamate from the transmitter pool (56). Additionally, it has been shown that the non-metabolizable glutamate analog d-aspartate is taken up into presynaptic nerve endings (57) as well as into cultured glutamatergic neurons with only a marginal astrocytic contamination (58). Altogether, these studies point to a quantitatively more important role of astrocytic glutamate uptake relative to that associated with neurons in the inactivation of transmitter glutamate. This notion is in keeping with the functional role of the glutamate-glutamine cycle and it fits with the model proposed by Hertz and Schousboe (59) based on analysis of kinetic data for glutamate uptake into cultured neurons and astrocytes.

### Role of GABA transporters for maintenance of GABA neurotransmission

The concept of termination of GABA mediated neurotransmission via high-affinity transport is based on pioneering studies by Elliott and Van Gelder (60) demonstrating that GABA was accumulated by cerebral cortical slices from the incubation medium against a concentration gradient. The high-affinity nature of the transport mechanism was demonstrated by Iversen and Neal (61) also using brain slices and this study showed GABA uptake in both neurons and astrocytes. Detailed kinetic studies of GABA uptake in cultured neurons and astrocytes [for review, see Ref. (62, 63)] have shown that the neuronal GABA uptake is more efficacious than that in astrocytes as schematically illustrated by Hertz and Schousboe (59). Hence, only 10–20% of synaptically released GABA will be taken up into astrocytic processes whereas the remaining GABA is re-accumulated in the presynaptic nerve endings of the GABAergic neurons where it may be re-utilized as a neurotransmitter after packaging in vesicles (64). This is in contrast to the scenario described above for glutamatergic neurotransmission in which the majority of the released transmitter is taken up into astrocytes which ensheathe the synapse.

The high-affinity GABA transporters have been cloned and four distinct transport proteins have been described three of which are specific for GABA and one is able to transport both GABA and the osmolyte betaine [for references, see Ref. (38)]. The nomenclature of these transporters is unfortunately somewhat confusing but it is recommended to utilize the HUGO nomenclature, i.e., GAT1, GAT2, and GAT3 for the GABA specific transporters and BGT1 for the betaine-GABA transporter (38, 65, 66). Among these transporters, GAT1 is the most prevalent being expressed both in GABAergic neurons and in astrocytes. GAT2 is expressed neonatally in the brain but its expression decreases as a function of postnatal development. GAT3 may be considered primarily astrocytic while BGT1 is only sparsely expressed in the brain [for references, see Ref. (38)]. While pharmacological studies have pointed to an interesting function of BGT1 and possibly GAT3 for the control of the availability of GABA at extrasynaptic GABA receptors (67) the low expression level of BGT1 has questioned the quantitative significance of this transporter for GABA homeostasis (68).

The fact that aberrations in GABA neurotransmission are associated with a number of neurological diseases including epilepsy [see Ref. (34) for references] has prompted detailed pharmacological studies of GABA transporters to be performed [for references, see Ref. (66)]. This has resulted in development of the clinically active antiepileptic drug tiagabine which is a specific inhibitor of GAT1 (34) and more recently such studies have been focusing on development of inhibitors acting preferentially on non-GAT1 transporters (69). This has led to synthesis of a GABA analog which preferentially inhibits BGT1 and which exhibits anticonvulsant activity (70). This is an indication that astrocytic GABA transport may represent an interesting pharmacological target as proposed previously (71, 72).

### Ammonia homeostasis related to the glutamate-glutamine cycle and shuttling of nitrogen between neurons and astrocytes

As seen in Figure 4 the glutamate-glutamine cycle leads to production of ammonia in glutamatergic neurons by the conversion of glutamine to glutamate catalyzed by PAG. At the same time ammonia is needed in the astrocytes for the synthesis of glutamine from glutamate catalyzed by GS. This obviously requires a mechanism by which the ammonium ion can be transported out of the neurons and into the surrounding astrocytes. While ammonia (NH_3_) may be able to diffuse through the plasma membrane it is less likely that the charged ammonium (${\mathrm{NH}}_{4}^{+}$) ion can penetrate the lipid double layer of these membranes and at physiological pH essentially all ammonia is in the ionized form of ammonium. It has therefore been proposed that an amino acid based shuttle mechanism may be operating between the neuronal and the astrocytic compartments [see Ref. (14)]. The operation of one of the models is based on the use of alanine as the nitrogen-carrier (13, 73) while the other analogous model has proposed that branched chain amino acids (BCAAs) and their congeneric keto-acids play this role (74). Regardless of the model there is a requirement for a reductive amination of α-ketoglutarate to glutamate catalyzed by GDH to take place with a simultaneous transamination of this glutamate to either alanine of a BCAA to occur [see Ref. (11) for details]. As the thermodynamic equilibrium of the GDH-catalyzed reaction and the high *K*_m_ value for [${\mathrm{NH}}_{4}^{+}$ of GDH counteract such amination (75)] it has been proposed that the intra-mitochondrial milieu possibly coupled to a metabolomic complex between PAG, GDH, and the transaminases in question, i.e., ALAT and BCAA-aminotransferase may facilitate the reactions (11). Actually, a metabolomic coupling between GDH and ALAT as well as BCAA-aminotransferase has been experimentally demonstrated (76, 77).

A recent review paper by Rothman et al. (78) has provided a careful analysis of the functional capability of the above mentioned shuttles to support the flux of nitrogen from neurons to astrocytes needed to balance the rate of *de novo* synthesis of glutamine to compensate for oxidative metabolism of glutamate in the astrocytic compartment. While the rate of ammonia fixation by the GDH reaction and the activity of the transaminases involved in the proposed shuttles may be sufficient to match the rate of glutamine synthesis, the modeling studies show that the actual fluxes of either the alanine/lactate or the BCAA/branched chain keto-acid shuttles are insufficient to account for the need of ammonia nitrogen to account for glutamine synthesis. Hence, the exact mechanisms involved in ammonia nitrogen transfer between the neuronal and astrocytic compartments remain to be fully elucidated.

### Energy substrates for the operation of glutamate/GABA cycling. Glycogen, glucose, lactate, or glutamate

#### Energy cost related to cycling

Since the transporters for glutamate and GABA are dependent on an intact trans-membrane sodium ion gradient maintained by the operation of the Na^+^-K^+^-ATPase it is clear that transport of the amino acids is strongly dependent on chemical energy supplied by ATP hydrolysis [see Ref. (43)]. Additionally, in case glutamate transported into astrocytes is metabolized to glutamine one molecule of ATP is used per glutamate in the GS catalyzed reaction. As will be discussed below, glutamate may alternatively function as an energy producing molecule in case it is oxidatively metabolized via the GDH-catalyzed reaction and subsequent metabolism of the carbon skeleton to CO_2_ in the TCA cycle. However, since glucose is the most important energy fuel for the brain under normal physiological conditions (43), the following sections shall deal with glucose and its associated molecules glycogen and lactate as energy sources. Glutamate as an energy source will be discussed separately.

#### Glycogen as energy source

**Figure 7:**
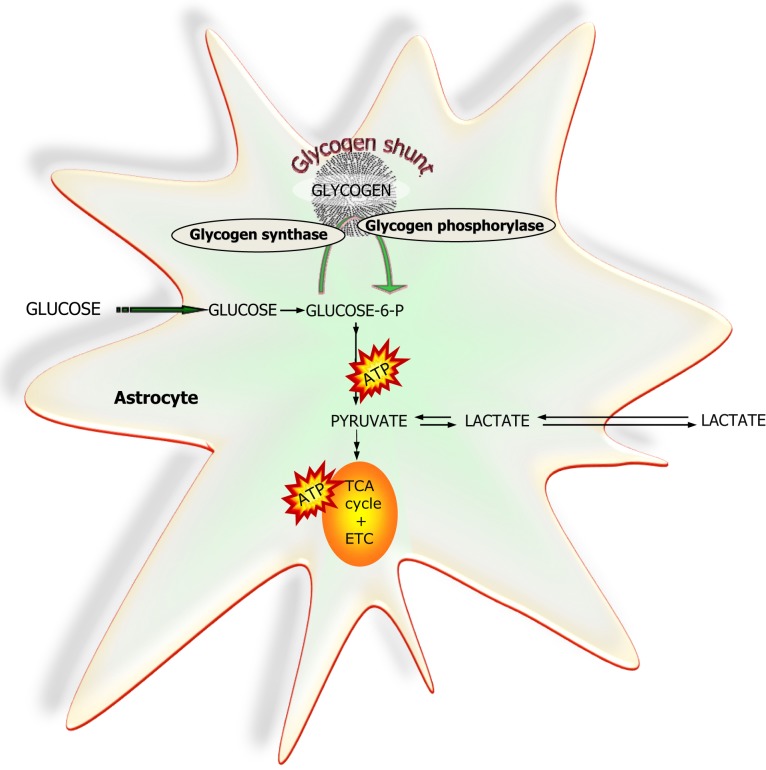
**A simplified schematic representation of glucose metabolism via glycolysis or via the “glycogen-shunt” illustrating how glucose units may be metabolized *via* incorporation into and subsequent hydrolysis from the branched glycogen molecule preceding metabolism to pyruvate and lactate, i.e., glycogenolysis**. Glucose-6-P, glucose-6-phosphate; TCA, tricarboxylic acid; ETC, electron transport chain.

The brain has a small but apparently functionally significant store of glycogen which is selectively located in the astrocytes [Ref. (79) and references herein]. It can be used during an energy crisis, i.e., reduced blood supply and/or aglycemia but recent studies have provided evidence that glycogen is a highly dynamic molecule which via the glycogen-shunt (Figure 7) channels a significant part of the glucose carbon entering the brain into glycolysis (79, 80). Studies utilizing drugs selectively inhibiting glycogen phosphorylase, the key enzyme in glycogenolysis have convincingly demonstrated that a continuous functional activity of the glycogen-shunt is a prerequisite for the maintenance of optimal glutamatergic activity and for functional and behavioral activities associated with such neurotransmission including memory formation and consolidation [for references, see Ref. (79) and (81)]. This obviously places the astrocytes in a key functional role since as mentioned above glycogen is exclusively found in astrocytes.

#### Glucose versus lactate as energy source

Whether or not glucose is metabolized through the glycogen-shunt its carbon skeleton enters the glycolytic metabolic pathway as glucose-6-phosphate and through a series of reactions referred to as the glycolytic pathway, the glucose molecule consisting of six carbon atoms is split into two molecules of pyruvate, i.e., two C-3 units (43). This results in a net production of two molecules of ATP if the glycogen-shunt is not operating since the hexokinase step consumes an ATP while three molecules of ATP are gained when the glycogen-shunt is in operation since the hexokinase step is circumvented. Therefore from a purely energetic efficiency point of view there is a dramatic advantage of the operation of the glycogen-shunt increasing the energy production of the glycolytic pathway by 50%. The two moles of pyruvate formed in glycolysis from glucose can either be converted to two moles of lactate or they can be oxidized to CO_2_ in the pyruvate dehydrogenase (PDH)/TCA cycle reactions. In the latter case the NADH + H^+^ produced in the oxidative processes of the glycolytic pathway are available for oxidative phosphorylation in the mitochondrial respiratory chain while in the former case the reduced co-enzyme is oxidized in the lactate dehydrogenase (LDH) catalyzed conversion of pyruvate to lactate [see Ref. (43) for further details]. It must be emphasized that in case pyruvate is to enter the PDH/TCA cycle pathway a mechanism capable of transferring reduced co-enzyme equivalents from the cytoplasm to the mitochondria and vice versa must exist or else the cytoplasmic content of NAD^+^ will be depleted. The shuttling mechanism for transfer of reduced co-enzyme equivalents across the mitochondrial membrane is of fundamental importance and the most important mechanism for this in the brain is the MAS discussed above in relation to the biosynthetic pathway for transmitter glutamate (43). While there is consensus about the presence of the MAS in neurons its expression level in astrocytes has been questioned (82). It should be noted, however, that a transcriptomic analysis of acutely isolated astrocytes has provided evidence that this shuttle is present in astrocytes (9, 83) which is in keeping with the high expression of all enzymes necessary for oxidation of glucose and the actual functional activity of complete glucose oxidation in these cells (9, 84).

The hypothesis proposed by Pellerin and Magistretti (85) that glutamate recycling in the glutamate-glutamine cycle is tightly coupled to astrocytic glycolysis converting glucose essentially quantitatively to lactate and that this lactate is transferred to neurons for complete oxidation [the astrocyte-neuron-lactate-shuttle (ANLS) has been a subject of considerable debate (84, 86, 87)]. The fact that astrocytes have a considerable oxidative metabolism of glucose (84) and that lactate can be transferred bi-directionally between neurons and astrocytes (88–91) is not in favor of ANLS being the unifying hypothesis of glucose metabolism and transfer of lactate to neurons to serve as the major energy substrate in these cells. Actually, it has been shown that glucose and not lactate is required to maintain glutamatergic activity in cultured cerebellar granule cells, a glutamatergic neuronal preparation (92–94). Additionally, Zielke et al. (95) have elegantly demonstrated that at least half of the extracellularly available glucose in the brain is accumulated by neurons and that lactate is metabolized by both neurons and astrocytes.

#### Glutamate as an energy substrate

The demonstration that glutamate can be oxidatively metabolized to CO_2_ in cultured astrocytes (18, 96, 97) provides strong support to the notion that the glutamate-glutamine cycle cannot operate in a stoichiometric fashion, i.e., that there is a 1:1 exchange of glutamate and glutamine (Figure 4). It is, however, compatible with a considerable pyruvate carboxylation enabling *de novo* synthesis of glutamine to compensate for loss of glutamate by oxidative metabolism (see above). That the carbon skeleton of glutamate to a considerable extent gets access to the TCA cycle can be deduced from experiments using [^13^C]glutamate and NMR spectroscopy to study glutamate metabolism in cultured astrocytes. It could be demonstrated that glutamate-carbon labeled lactate derived from the TCA cycle via ME activity more extensively than glutamine formed in the GS catalyzed reaction (98). It should be emphasized that complete oxidation of glutamate via the combined action of the GDH reaction, pyruvate recycling and the TCA cycle generates 75% of the energy in the form of ATP that would be produced by oxidation of glucose. The significance of glutamate as an energy source is further discussed by Kreft et al. (81).

## Concluding Remarks

A recent comprehensive review on astrocyte physiology and pathophysiology (99) has provided compelling evidence to support the view which has emerged over the past several years that these cells constitute as aptly put by Hertz and Zielke (100): “The stars of the show.” The present review has focused on the functional role of astrocytes in relation to their regulatory function in amino acid neurotransmission. The main conclusion is that these neurotransmission processes which operate in the vast majority of the synapses in the brain would be completely dysfunctional without astrocytic support and regulatory control. In this context it is important to note that astrocyte morphology and process ramification has undergone a dramatic increase in sophistication from small rodents to humans which by far exceeds the corresponding difference seen for nerve endings (101).

## Conflict of Interest Statement

The authors declare that the research was conducted in the absence of any commercial or financial relationships that could be construed as a potential conflict of interest.
